# Performance of the Severe Acute Respiratory Illness Sentinel Surveillance System in Yemen: Mixed Methods Evaluation Study

**DOI:** 10.2196/27621

**Published:** 2021-07-09

**Authors:** Ghamdan Gamal Alkholidy, Labiba Saeed Anam, Ali Hamoud Almahaqri, Yousef Khader

**Affiliations:** 1 Yemen Field Epidemiology Training Program Ministry of Public Health and Population Sana'a Yemen; 2 National Influenza Control Program Ministry of Public Health and Population Sana'a Yemen; 3 Department of Community Medicine, Public Health and Family Medicine Faculty of Medicine Jordan University of Science & Technology Irbidjord Jordan

**Keywords:** evaluation, surveillance, Centers for Disease Control and Prevention guidelines, severe acute respiratory illness, Yemen

## Abstract

**Background:**

The national severe acute respiratory illness (SARI) surveillance system in Yemen was established in 2010 to monitor SARI occurrence in humans and provide a foundation for detecting SARI outbreaks.

**Objective:**

To ensure that the objectives of national surveillance are being met, this study aimed to examine the level of usefulness and the performance of the SARI surveillance system in Yemen.

**Methods:**

The updated Centers for Disease Control and Prevention guidelines were used for the purposes of our evaluation. Related documents and reports were reviewed. Data were collected from 4 central-level managers and stakeholders and from 10 focal points at 4 sentinel sites by using a semistructured questionnaire. For each attribute, percent scores were calculated and ranked as follows: very poor (≤20%), poor (20%-40%), average (40%-60%), good (60%-80%), and excellent (>80%).

**Results:**

As rated by the evaluators, the SARI surveillance system achieved its objectives. The system’s flexibility (percent score: 86%) and acceptability (percent score: 82%) were rated as “excellent,” and simplicity (percent score: 74%) and stability (percent score: 75%) were rated as “good.” The percent score for timeliness was 23% in 2018, which indicated poor timeliness. The overall data quality percent score of the SARI system was 98.5%. Despite its many strengths, the SARI system has some weaknesses. For example, it depends on irregular external financial support.

**Conclusions:**

The SARI surveillance system was useful in estimating morbidity and mortality, monitoring the trends of the disease, and promoting research for informing prevention and control measures. The overall performance of the SARI surveillance system was good. We recommend expanding the system by promoting private health facilities’ (eg, private hospitals and private health centers) engagement in SARI surveillance, establishing an electronic database at central and peripheral sites, and providing the National Central Public Health Laboratory with the reagents needed for disease confirmation.

## Introduction

Worldwide, acute lower respiratory infection is the second commonest cause of morbidity and the third commonest cause of mortality in all age groups [1]. A significant proportion of the global burden of acute lower respiratory infection, especially in children and older adults, is attributable to influenza and respiratory syncytial viruses.

The World Health Organization (WHO) estimated that worldwide annual influenza epidemics result in about 3 million to 5 million cases of severe illness and about 250,000 to 500,000 deaths. In early 2019, the Global Burden of Disease study estimated that 99,000 to 200,000 annual deaths resulting from lower respiratory tract infections are directly attributable to influenza [1,2].

Estimates are rare in many countries, including countries in the Eastern Mediterranean Region. The influenza A (H1N1) pandemic highlighted the necessity of reliable estimates for the disease burden of severe acute respiratory illness (SARI) and influenza-associated SARI (F-SARI) in all countries and regions of the world [3].

Many countries have established sentinel sites for influenza epidemiological surveillance. The data captured from sentinel sites have been used by WHO member states to estimate disease burden at the national level and to compare data between countries.

Due to the fact that many emerging and reemerging diseases classified under the International Health Regulations are of an acute respiratory nature (eg, SARS [severe acute respiratory syndrome], MERS-CoV [Middle East respiratory syndrome coronavirus], and novel influenza pathogens such as H5N1 and H7N7), it is necessary to strengthen surveillance systems for acute respiratory infections and influenza in all WHO member states. This will enable countries to produce more accurate estimations of SARI and F-SARI burden [1,2].

Based on the Worldometer elaboration of the latest United Nations data in 2020, the current population size of Yemen is 29,771,764 [4]. Yemen has 4 seasons, but it is likely that the influenza virus is being circulated throughout the year. Thus, there is a great probability that different patterns of influenza virus circulation occur throughout the year [5]. Yemen is one of the countries that experience a high number of deaths resulting from acute and chronic respiratory infections. In 2008, the Ministry of Public Health and Population initiated the National Influenza Control Program to enhance the country’s capacity in monitoring influenza diseases among community, guide the country in reducing morbidity and mortality from influenza diseases through the early detection of emerging novel influenza subtypes, provide a timely response for influenza prevention and control, and provide recommendations for improving influenza surveillance.

In 2013, more than 250 cases of influenza and 10 deaths were reported. A total of 1811 patients with SARI were admitted from 2011 to 2016. Of these patients, 1413 (78%) were aged <15 years, 89 (4.9%) patients had influenza viruses, and 655 (36.2%) had noninfluenza viruses. Further, the case fatality rate was 8% [6].

The Surveillance Department of the Ministry of Public Health proposed the inclusion of influenza in the Joint Program Review and Planning Mission [6]. The Central Public Health Laboratory was recognized as a national influenza center where facilities for carrying out polymerase chain reaction and serology tests are available and functional [6,7].

The WHO recommends that countries should perform surveillance for SARI and F-SARI and that surveillance systems should undergo periodic comprehensive evaluations. To ensure that the objectives of national surveillance are being met, this study aimed to examine the level of usefulness and the performance of the SARI surveillance system.

## Methods

### Study Design

A descriptive study was conducted to evaluate the SARI surveillance system from October to December 2018 based on the updated Centers for Disease Control and Prevention guidelines for the evaluation of a public health surveillance system [8]. Mixed methods with quantitative and qualitative components were used for the evaluation. SARI sentinel sites at four public hospitals—Al Joumhouri, Al Wahda, Al Swaide, and Al Thawra—in four governorates (Sana’a city, Aden, Taiz, and Al Hodeida) were studied. All possible stakeholders, including National Influenza Control Program managers, data entry staff, Ministry of Public Health and Population staff, and members of focal points in sentinel sites, were enrolled in this study.

### Data Collection

A desk review of all documents, guidelines, strategies, and pertinent scientific literature on influenza programs was conducted. Data were collected via in-depth interviews and semistructured questionnaires (Multimedia Appendix 1) with stakeholders and members of focal points at the sentinel sites, respectively. In addition, a review of the SARI system database was conducted.

### System Attributes

A total of 9 surveillance system attributes that can affect usefulness were assessed. Quantitative analysis was used to assess data quality, timeliness, sensitivity, and positive predictive values. Qualitative analysis was used to assess representativeness, simplicity, flexibility, acceptability, and stability.

### Analysis Methods

To determine the level of usefulness, the system was considered useful if it met at least one of its objectives and one of its planned uses. With regard to qualitative attributes, stakeholders were asked to rate the degree to which they agreed with attributes’ specific indicators by using a 5-point Likert scale (1=strongly disagree; 2=disagree; 3=neutral; 4=agree; 5=strongly agree). Higher scores indicated better performance in terms of the studied attribute. The scores of all indicators for each attribute were summed and divided by the maximum scores to produce a percent score. The percent score was used to rank each attribute. The final rank of each attribute was classified as follows: excellent (attribute score: >80%), good (attribute score: 60%-80%), average (attribute score: 40%-60%), poor (attribute score: 20%-40%), and very poor (attribute score: ≤20%).

With regard to quantitative attributes, data quality was assessed by measuring the completeness of patient interview forms, form transmission data, and respiratory specimen collection and testing data and by checking signs and symptoms records and primary diagnoses to determine whether surveillance case definitions had been adhered to properly. The data collected by the system were compared against the minimum data collection standards for SARI surveillance. Timeliness was assessed by calculating the percentage of specimens that were collected and sent to the laboratory within 72 hours.

## Results

### Findings From the Desk Review for Describing the SARI System

#### The Main Purpose and Objectives of the SARI System

The National Influenza Sentinel Surveillance system was established in 2010. The main purpose and objectives of the SARI system are to monitor influenza occurrence in humans and to provide a foundation for detecting outbreaks of novel strains of influenza.

#### SARI Case Definition

A case of SARI was defined as a person meeting the case definition of influenza-like illness (ie, the sudden onset of a fever of >38 °C and at least 1 of the following respiratory symptoms: dry cough, sore throat in the absence of another diagnosis, and shortness of breath or difficulty in breathing requiring hospital admission).

#### Sources of Data for the SARI System

Surveillance for influenza-like illness and SARI was carried out in 4 sentinel sites. Aggregated data were collected from and reported by each sentinel site. The data included the following:

- the number of new SARI cases during the reported week
- the number of new SARI cases in which specimens were collected during the reported week
- the total number of new hospital admissions to wards in which SARI surveillance is being conducted
- specimen and epidemiological data

Specimens and epidemiological data are collected from the sentinel sites and transported to national public health laboratories. At the laboratory, specimens are tested for the influenza A and B viruses and are further subtyped if they test positive for the influenza A virus. Epidemiological and virological data collected from the sentinel sites should be collected and reported regularly to the national health authorities on a weekly basis throughout the year.

#### Data Flow and Feedback in the SARI Surveillance System

**Figure 1:**
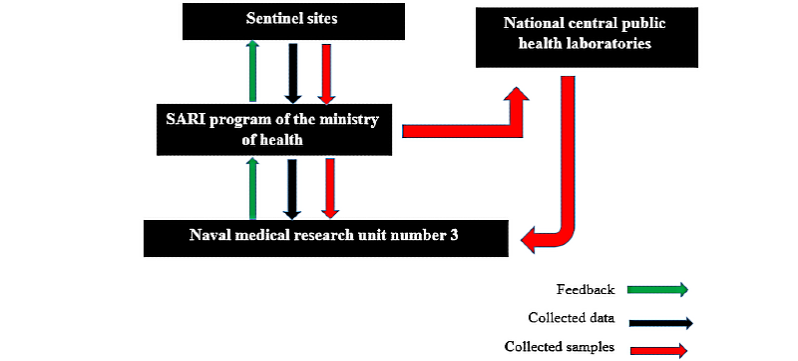
This figure illustrates the data flow and feedback in the influenza-like illness and SARI surveillance program of the Ministry of Public Health and Population in Yemen. SARI: severe acute respiratory illness.

The system was designed so that each sentinel site could send its reports to central sites within 1 week. Further, samples sent to National Central Public Health laboratories within 72 hours are sent to US Naval Medical Research Unit Number 3 for confirmation, as shown in Figure 1. At the central sites, data are reviewed, organized, and analyzed, as required. Feedback is then sent to sentinel sites.

### Demographic Characteristics of the Participants

A total of 3 persons at the central sites and 10 persons at the peripheral sites (3 pediatricians, 3 nurses, 2 laboratorian specialists, 3 health system directors, and 2 medical specialists) evaluated the system. The mean age of the participants was 44.5 years.

### Findings From In-depth Interviews at Central and Peripheral Sites

#### Usefulness

Of the 4 SARI stakeholders at the central sites, 3 (75%) agreed that the SARI surveillance system met its objectives. The usefulness percent score was 75%, indicating that the usefulness of the SARI system was good.

#### Flexibility

All 13 respondents agreed that the system could easily adapt to changes in the SARI case definition, include other diseases, and accommodate any changes in data with less effort and minimal costs. The overall percent score for flexibility was 86%, which indicated excellent performance (Table 1). At sentinel sites, the percent score, as determined by 10 stakeholders, was 71%. This indicated that the system had good performance at the peripheral sites.

**Table 1:** Flexibility of the severe acute respiratory illness (SARI) surveillance system (N=13).^a^

Indicator	Total score	Percent score	Rank
The system could adapt easily to changes in the SARI case definition	34	83	Excellent
The system can adapt to the integration of other surveillance systems	35	84	Excellent
The system adapted to accommodate new, additional information (eg, variation in resources)	41	90	Excellent

^a^The total score, percent score, and rank of the system were 120, 86, and excellent, respectively.

#### Stability

All 4 stakeholders at the central sites agreed that the system could adapt to changes in resources, but half of the stakeholders (2/4, 50%) stated that the system mainly depends on external funds. With regard to the scoring system, the overall stability percent score was 75% (Table 2). This indicated that the stability of the SARI system was good.

**Table 2:** Stability of the severe acute respiratory illness system (n=4).^a^

Indicator	Total score	Percent score	Rank
The system can adapt to changes in resources	4	100	Excellent
The system can adapt to funding withdrawal	2	50	Average

^a^The total score, percent score, and rank of the system were 6, 75, and good, respectively.

### Findings From the Self-Administered Semistructured Questionnaire for Sentinel Site Focal Points

#### Simplicity

In total, 2 indicators of simplicity were ranked as excellent, 3 were ranked as good, and 1 was ranked as average. The simplicity percent score of the system was 74% (Table 3), indicating that the SARI system’s simplicity attribute was good.

**Table 3:** Simplicity of the severe acute respiratory illness (SARI) system based on total scores, percent scores, and rank.^a^

Indicator	Total score	Percent score	Rank
There is the existence of a SARI case definition	30	60	Average
Using the SARI case definition is easy	37	74	Good
The SARI system uses an easy and understandable format	37	74	Good
Writing a SARI report does not take much time	40	80	Excellent
The trainees had training	42	80	Excellent

^a^The total score, percent score, and rank of the system were 186, 74, and good, respectively.

#### Acceptability

The two indicators of acceptability (ie, the willingness to participate among people within the system and satisfaction with the SARI surveillance system) were ranked as excellent. The acceptability percent score of the SARI system was 82% (ie, the SARI system had excellent performance; Table 4).

**Table 4:** Acceptability of the severe acute respiratory illness (SARI) surveillance system among sentinel sites (n=10).^a^

Indicator	Total score	Percent score	Rank
Are you willing to participate within the system?	40	80	Excellent
Are you satisfied with the SARI surveillance system?	42	84	Excellent

^a^The total score, percent score, and rank of the system were 82, 82, and excellent, respectively.

### Findings From the Review of the SARI System Database

#### Data Quality

Data quality was evaluated by assessing the percentage of complete forms and the missing variable data in the forms. All of the patients included in the central sites’ database (N=245) had complete forms (245 forms; 100% completeness). With regard to missing data, 22 case report forms from 2018 were selected randomly and reviewed. No missing variables were found, and the variables in these forms were in line with those of the database (completeness: 100%; accuracy: 97%). The overall data quality percent score was 98.5% (excellent).

#### Timeliness

The percentage of collected specimens at the health facilities that sent samples to the laboratory within 72 hours was used as an indicator of timeliness. Of the 182 collected samples, 42 (23.1%) samples were sent to the laboratory within 72 hours. The percent score for timeliness was 23% in 2018, indicating that the system’s timeliness was poor.

### Overall Performance of the SARI Surveillance System

The overall performance of the SARI surveillance system had a percent score of 79% (ie, the SARI system had good performance; Table 5).

**Table 5:** The overall performance of the severe acute respiratory illness system in Yemen.

Indicators	Score	Percent score	Rank
Usefulness	15	75	Good
Flexibility	120	86	Excellent
Stability	6	75	Good
Simplicity	186	74	Good
Acceptability	82	82	Excellent
Timeliness	23	23	Poor
Data quality	99	99	Excellent
Overall performance	394	79	Good

### Strengths and Weaknesses of the SARI Surveillance System

Despite its many strengths, the SARI system has some weaknesses. For example, it depends on irregular external financial support. To act as a platform for the surveillance of other respiratory illnesses, the SARI surveillance system integrates an influenza-like illness surveillance system with the electronic Disease Early Warning System. This has several benefits. First, it allows for efficient laboratory data collection and transportation. Second, the SARI system uses resources more efficiently than other systems. These widespread benefits enhance the usefulness of the system and allow the system to meet its own surveillance objectives and address broader national priorities.

## Discussion

### Principal Findings

The surveillance systems at sentinel sites are tools for the early detection of disease, the monitoring of trends in the burden of diseases, and the generation of recommendations for the prevention and control of diseases. The evaluation of surveillance systems helps decision makers to set priorities for future planning, resource allocation, and future interventions for preventing the spread of diseases.

Overall, this study showed that the performance of the SARI surveillance system was good. Studies from Zambia [9] and the Democratic Republic of Congo [10] have reported similar findings. The SARI system was found to be useful in detecting trends and signal changes in the occurrence of SARI, estimating the magnitude of morbidity and mortality related to SARI, and promoting research for informing prevention and control measures for SARI. Similarly, a previous evaluation in Zambia demonstrated the usefulness of the system [9].

The SARI surveillance system was shown to be simple. The case definition and case report forms were available and easy to use. Similarly, the simplicity of the SARI surveillance system was documented in previous evaluations conducted in the Democratic Republic of Congo [10] and Zambia [10].

The SARI surveillance system’s flexibility was excellent. The system appeared to be able to adapt easily to changes in the SARI case definition and accommodate changes in data with less effort and minimal costs. This finding is consistent with the findings of a study from South Africa [11]. However, it is not consistent with the findings of studies from Zambia [9] and the Democratic Republic of Congo [10], which reported that the flexibility of the evaluated systems ranged from moderate to good. The acceptability of the SARI system was excellent, as reflected by the willingness of stakeholders to participate in the system and their satisfaction with the SARI surveillance system.

The stability of the SARI surveillance system was good. It was found that the system was stable and could adapt to changes in resources (eg, donors withdrawing their support). This finding is in line with the findings of previous studies from South Africa [11], the Democratic Republic of Congo [10], and Zambia [9]. However, it is not in line with the findings of a study from Pakistan [12], which reported average stability.

Our findings showed that the timeliness of the SARI system was very poor. This might be due to the lack of laboratory components that are essential for sampling. Previous evaluations in South Africa [11], China [13], Zambia [9], and the Democratic Republic of Congo [10] reported moderate to good timeliness. The quality and completeness of SARI surveillance system data were excellent.

### Limitations

We could not calculate positive predictive values and assess sensitivity because the samples have not been tested since 2016 due to a lack of reagents.

### Conclusion

Overall, the SARI surveillance system was useful in estimating morbidity and mortality, monitoring the trends of the disease, and promoting research for informing prevention and control measures. The overall performance of the SARI surveillance system was good. We recommended expanding the system by promoting private health facilities’ (eg, private hospitals and private health centers) engagement in SARI surveillance, establishing an electronic database at central and peripheral sites, and providing the National Central Public Health Laboratory with the reagents needed for disease confirmation.

## Appendix

Multimedia Appendix 1 Questionnaire.

## Abbreviations

F-SARI: influenza-associated severe acute respiratory illness
MERS-CoV: Middle East respiratory syndrome coronavirus
SARI: severe acute respiratory illness
SARS: severe acute respiratory syndrome
WHO: World Health Organization
