# Supplementary material for: Performance of the Severe Acute Respiratory Illness Sentinel Surveillance System in Yemen: Mixed Methods Evaluation Study
Source: JMIR Public Health Surveill. 2021 Jul 9;7(7):e27621. doi: 10.2196/27621 (PMC8304118; doi:10.2196/27621)
Supplement: Multimedia Appendix 1 [file publichealth_v7i7e27621_app1.docx]

**Multimedia appendix**

**Performance of Severe Respiratory Illness Sentinel Surveillance System in Yemen: Evaluation Study**

**A: (Central level, questionnaire)**

**No of Q:** _____________ **Age:** ________  **Sex: __________**

**Job:** ____________ **Date**: ________

1. **Usefulness**

**The system provide estimates of the magnitude of morbidity and mortality related to SARI disease?**

0-No 1-Yes

**The system stimulate researches to inform prevention and control measures of SARI disease?**

0-No 1-Yes

**The system permit planning the resources for prevention, control and interventions activities?**

0-No 1-Yes

**Does the system’s databased is using to update & develop the national policy strategy about SARI disease?**

0-No 1-Yes

**The system detects trends that signal changes in the occurrence of SARI disease, including detection of epidemics (or outbreaks).**

0-No 1-Yes

**The system detects SARI cases in a timely way to permit accurate diagnosis and identification of causative organism?**

0-No 1-Yes

**Do you have any comments?**

…………………………………………………………………………………….

1. **Flexibility**

**Does the system adapted to accommodate to new additional information (e.g. change in case definition)?**

0-No 1-Yes

**Does the system adopt to integrate with other surveillance?**

0-No 1-Yes

**Does the system adapted to accommodate to new health related events with little resources and time?**

0-No 1-Yes

**Do you have any comments?**

…………………………………………………………………………………….

1. **Stability**

**Does the system have an external fund?**

0-No 1-Yes

**Does the system have governmental fund?**

0-No 1-Yes

**Do you think the system is stable without sponsors fund?**

0-No 1-Yes

**Do you think the system not required time to collecting, sending, receiving and manage data?**

0-No 1-Yes

**Do you have any comments?**

…………………………………………………………………………………….

**Thank you very much for your participating and answering**

**B: (Governorates and health facilities levels, questionnaire)**

**No of Q:** _____________ **Age:** ________  **Sex: __________**

**Job:** ____________ **Date**: ________

1. **Simplicity**

**The standard case definition of SARI case is existing in the facility?**

Strongly disagree Disagree Natural Agreed Strongly agree

**The case definition easy to be applied?**

Strongly disagree Disagree Natural Agreed Strongly agree

**The formats are clear & easy to be filled?**

Strongly disagree Disagree Natural Agreed Strongly agree

**The staff had a special training to update, use the system?**

Strongly disagree Disagree Natural Agreed Strongly agree

Do you have any comments?

…………………………………………………………………………………….

1. **Flexibility**

**The system can adapt to accommodate to new changes in case definition?**

Strongly disagree Disagree Natural Agree Strongly agree

**The system adapts to accommodate to new introduction of data from other system?**

Strongly disagree Disagree Natural Agree Strongly agree

**The system adapts to accommodate to new variation in resources?**

Strongly disagree Disagree Natural Agree Strongly agree

**The system adapted to accommodate to new health related events with little resources and time?**

Strongly disagree Disagree Natural Agree Strongly agree

**Do you have any comments?**

…………………………………………………………………………………….

1. **Acceptability**

**You are willing to participate within the system.**

Strongly disagree Disagree Natural Agree Strongly agree

**The staff satisfied with program system.**

Strongly disagree Disagree Natural Agree Strongly agree

**Do you have any comments?**

…………………………………………………………………………………….

1. **Stability**

**How many times the SARI surveillance system is stopped to investigate in last months?**

1. One time
2. Two times
3. Many times

**Do you have any comments?**

…………………………………………………………………………………….

**Thank you very much for your participating and answering**
